# Bacterial Chemotaxis: Introverted or Extroverted? A Comparison of the Advantages and Disadvantages of Basic Forms of Metabolism-Based and Metabolism-Independent Behavior Using a Computational Model

**DOI:** 10.1371/journal.pone.0063617

**Published:** 2013-05-22

**Authors:** Matthew D. Egbert

**Affiliations:** 1 Biosystems Analysis Group, Friedrich Schiller University, Jena, Germany; 2 Centre for Computational Neuroscience and Robotics, University of Sussex, Brighton, United Kingdom; University of Westminster, United Kingdom

## Abstract

Using a minimal model of metabolism, we examine the limitations of behavior that is (a) solely in response to environmental phenomena or (b) solely in response to metabolic dynamics, showing that basic forms of each of these kinds of behavior are incapable of driving survival-prolonging behavior in certain situations. Inspired by experimental evidence of concurrent metabolism-based and metabolism-independent chemotactic mechanisms in *Escherichia coli* and *Rhodobacter sphaeroides*, we then investigate how metabolism-independent and metabolism-based sensitivities can be integrated into a single behavioral response, demonstrating that a simple switching mechanism can be sufficient to effectively integrate metabolism-based and metabolism-independent behaviors. Finally, we use a spatial simulation of bacteria to show that the investigated forms of behavior produce different spatio-temporal patterns that are influenced by the metabolic-history of the bacteria. We suggest that these patterns could be a way to experimentally derive insight into the relationship between metabolism and chemotaxis in real bacteria.

## Introduction

Certain species of bacteria are capable of moving up or down chemical gradients in what as known as bacterial chemotaxis. Chemotaxis is one of the simplest behaviors known, and it likely is one of the first behaviors to have existed in the history of life on earth. It is therefore one of the best studied forms of behavior, with a line of research that dates back to some of the earliest microscopic observations of bacteria by Leeuwenhoek in the 17th century and continues today, employing some of the most modern computational and mathematical modeling tools[1]–[3]. Empirical work and theoretical modeling have led to a detailed understanding of many aspects of the chemotactic mechanism in various bacteria, with a particular focus on. In particular, advances have been made in understanding flagellar motors [4], transmembrane chemo-receptors [5], signal transduction pathways [6]–[8], and in how bacteria are capable of responding to a very wide range of stimulus levels [9]–[11].

Some bacteria, such as *Pseudomonada* [12] and *E.coil* [13], move toward certain attractants in a “metabolism-independent” form of chemotaxis, where their behavior is in direct response to environmental features, such as the local concentration of attractant. For other bacteria, such as, the dominant mechanism is a response to the state of the metabolism, in what is referred to as “metabolism-dependent” or “metabolism-based” chemotaxis [14]. Instead of responding directly to environmental phenomena, these bacteria respond to the concentration of a metabolic product or some other aspect of the metabolic machinery, such as the state of a metabolic intermediary in the electron-transport system [15], [16]. Evidence suggests that various bacteria including *E*.*coli* [13], [17], [18], and *Rhodobacter sphaeroides* [19] employ *both* metabolism-independent and metabolism-dependent mechanisms.

A fundamental question remains concerning why different organisms employ these different forms of chemotaxis. It remains unclear whether the variety of mechanism seen in nature is due to non-adaptive stochastic processes such as genetic drift, or if the two forms of behavior are better suited to particular environments, resulting in the evolution of different mechanisms in different species. It has not yet been established how metabolism-based and metabolism-independent behaviors might be integrated into a coherent, functional behavior to drive behavior that is superior than either single behavior on its own. Goldstein and Soyer [20] simulated the evolution of chemotaxis pathways in virtual bacteria to gain insight into how and why certain chemotaxis pathways may have evolved. They showed that a simple, non-adaptive, metabolism-independent mechanism can accomplish chemotaxis and (in their model) was more easily evolved than an adaptive form of chemotaxis. They also demonstrated that different environmental conditions can cause different types of chemotactic mechanism to evolve, but the authors commented that “Untangling the role of each type of dynamics in the efficiency of chemotaxis requires further detailed analyses.” [20, p.5]. The analysis presented in this paper also provides insight into why different organisms should employ different forms of chemotaxis, but here we take a different approach. Instead of simulating the evolution of chemotaxis, we use a dynamical model to compare four different forms of metabolism-independent and metabolism-based behavior. Unlike Goldstein’s model, we include metabolic dynamics in our simulation. This makes possible the comparison of metabolism-independent and metabolism-based responses, allowing us to develop an understanding of their advantages and limitations.

The chemotaxis mechanisms employed by modern bacteria are sophisticated and therefore complicated. Despite many years of research we still do not fully understand how they work. In this paper, we present an analysis of the dynamics of basic forms of metabolism-based and metabolism-independent behaviors. Understanding how these basic mechanisms work is a helpful step towards understanding the more complicated mechanisms employed by modern bacteria, and understanding how bacteria regulate their interaction with their environment should improve our ability to fight infections, engineer environments that allow for the culturing of a wider variety of bacteria, and eventually, to engineer similar mechanisms in synthetic protocells enabling forms of dynamic stability based on ongoing environmental interaction.

### 1.1 The Role of Metabolism in Bacterial Chemotaxis

In 1969, Julius Adler published a series of experiments that suggested that for, chemotaxis is metabolism-independent. He demonstrated that for that species, certain attractants are not metabolizable and conversely, that certain metabolizable chemicals do not act as attractants [13]. Since then, much has been done to elucidate how the metabolism-independent mechanism of works, including details of transmembrane receptors, the two-component signal transduction system and how these systems can influence flagellar rotation (for a review, see [21]). However, work previous to Adler’s studies [22] and a growing body of recent research indicate that, at least for some bacteria, metabolism plays an ongoing role of influencing chemotactic behavior (see [23], [24] for recent reviews). This metabolism-dependent behavior has been observed in various bacteria [14], [25]–[27], and in some cases appears to be the primary chemotactic mechanism [14]. Details of how these metabolism-dependent mechanisms operate are beginning to emerge, with evidence suggesting that at least in some cases, metabolism-dependent chemotaxis is driven by a sensitivity to changes in the electron transport system [15], [16].

There is also evidence to suggest that in addition to the metabolism-independent mechanisms in studied by Adler, metabolism-dependent mechanisms of chemotaxis may also be at work [17], [18]. This is interesting not only because it lies in tension with Adler’s original findings, but also because it suggests the existence of both forms of chemotaxis are at work within a single organism. Adding weight to this idea, that have been genetically “gutted” of their metabolism-independent chemotaxis machinery still perform a limited form of chemotaxis [28], [29], again suggesting that multiple, concurrent chemotaxis mechanisms are in operation.

Concurrent metabolism-independent and -dependent mechanisms also appear to be operating within *R. sphaeroides*, which has multiple chemoreceptor clusters; one in the cytoplasm and one at the cell pole, suggesting multiple chemo-sensory pathways [19]. In this species, taxis towards certain sugars requires metabolism of those sugars, suggesting that some chemotactic behavior in is “likely to be generated by metabolic intermediates or the activities of the electron-transport chain and not by a cell-surface receptor or the rate or mode of substrate transport” [30]. Hamadeh et al. [31] have used a computational model to test different relationships of feedback between the clusters and signaling enzymes and eliminate possible relationships between the clusters, leading them to the conclusion that for this species, the two chemotaxis pathways likely “initially evolved independently and then became part of the same organism by horizontal gene transfer.” [31, p. 8]. The role of metabolism, the receptor clusters and their relationship remains in need of further investigation as it remains unclear precisely what it is that the cytoplasmic receptor cluster senses [31, pps. 11–12]. The state of affairs also remains ambiguous for strains of *Pseudomonads*, for which taxis towards aromatics appears to be metabolism independent [12], but taxis towards (methyl)phenols is metabolism-dependent [32].

There is substantial evidence for both metabolism-independent and metabolism-based chemotaxis. This prompts questions about why we should expect to see both forms of chemotaxis in nature, and in which situations or environments we should expect to see each form. Put another way, the advantages and limitations of basic forms of metabolism-independent and metabolism-based behavior have yet to have been fully established. Is a simple, metabolism-independent regulation of environmental conditions sufficient to drive survival-prolonging behavior in all conditions? Conversely, can a purely metabolism-based behavior that does not respond directly to any environmental features consistently drive “correct” (i. e., survival-prolonging) behavior? If not, what kind of combination of metabolism-independent and metabolism-based responses is optimal? Must such a mechanism of integration be complicated to produce improved behavior, or could a simple mechanism of integration suffice? These are theoretical issues that are very difficult to study in vitro, but are well suited to analysis via minimalistic mathematical models. Thus, the model and its analysis presented below do not make specific predictions about the behavior of specific species of bacteria, but instead provide theoretical insight into the basic forms of metabolism-independent and metabolism-based behavior which will help guide the interpretation of experimental results, and the design of future experiments.

Our analysis takes the following form: to study the relationships between behavior and metabolism, we use a minimalistic mathematical model to evaluate and compare four different idealized forms of behavior, one metabolism-independent, two metabolism-based, and one that is a combination of metabolism-independent and metabolism-based responses. For each of the behaviors we (i) examine example trajectories to identify limitations of the behavior, (ii) quantify the set of metabolic and environmental conditions that are survived or not survived by the behavioral mechanism, and (iii) present a spatial simulation indicating how bacteria employing the different behaviors would respond to a chemical gradient. We first investigate the metabolism-independent and metabolism-based behaviors. We show that each of these three forms of behavior is incapable of driving survival-prolonging behavior in certain survivable initial conditions. This leads to the investigation of a behavior that switches between metabolism-based and metabolism-independent mechanism. In our model, this switching mechanism is capable of surviving more conditions that any of the three more simple behaviors, demonstrating that a simple switching mechanism can suffice in some conditions to combine metabolism-independent and metabolism-based behavior into an improved behavior, and providing theoretical support for the empirical evidence of metabolism-based and metabolism-independent mechanisms operating concurrently in bacteria such as *E. coli* and *R. sphaeroides*.

The next section describes the model we use to make our comparisons. The Results Section describes and presents the analysis of the four simulated behaviors and the discussion recapitulations the main points of the paper and discusses the details and implications of the results.

## Model

### 2.1 A Minimal Model of a Metabolism

To compare different forms of metabolism-independent and metabolism-based behavior we have designed a minimalistic and abstract model, similar to those that we have used to study the adaptivity provided by metabolism-based chemotaxis [33], the evolutionary advantages of metabolism-based chemotaxis [34], and how metabolism-based behavior forms a basis for understanding normative behavior and agency [35]. The model is not intended to be a detailed simulation of the metabolic dynamics of a specific organism. Instead, it is used to facilitate investigation of possible relationships between metabolism and behavior by (i) stimulating discussion about how metabolism and behavior can interact, (ii) demonstrating the types of conditions in which metabolism-based and metabolism-independent behavior can or cannot appropriately regulate environmental conditions, and (iii) enabling the formal description and analysis of hypothetical interactions between metabolism and behavior.

The model is therefore simple and abstract, involving the simulation of the concentration of two distinct categories of chemical: metabolites, labeled 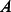, and resources or ‘food’ chemicals, labeled 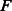. 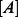 represents the concentration of all of the different chemicals in an autocatalytic metabolic network and 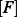 represents the concentration of any and all available resources that can be transformed by the metabolism into metabolites. The model involves some arbitrarily selected parameters, but as we shall see it nevertheless allows us to garner insight into possible relationships between metabolism and behavior, and to elaborate upon some general advantages and disadvantages of the two different forms of behavior. An alternative approach would have been to use one of the existing more detailed models of metabolism (e. g. a model of *E. coli* central metabolism [36]), but these are more complex, high-dimensional models with many parameters and they are, therefore, more difficult to understand and to visualize. More importantly, these more complex models do not include all of the details that are necessary for our analysis (in particular, the “viability limits” that we describe later in this section), meaning that even if we were to base our analysis on more realistic models of metabolism, we would have to include some arbitrarily parameterized viability limits. For these reasons, in this case, the advantages of using a simple model outweigh the advantages of using a more complex, detailed model.

Our analysis is decoupled from the details of the metabolic dynamics, while remaining connected to the general properties of metabolic systems, in particular, the view of metabolism as an autocatalytic, dissipative, far-from-equilibrium system that in the absence of (self-)construction and/or (self-)maintenance will naturally decay or degrade over time [37]–[39]. To capture these properties, we simulate (i) an autocatalytic transformation of 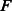 into 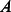 (Equation 1) and (ii) the collection of processes through which 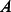, degrades, diffuses away from the system, or in some other way is transformed such that it has no subsequent influence on the system (Equation 2).
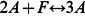
(1)

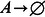
(2)


Starting from these two equations, we derived the following ordinary differential equation that describes the intrinsic metabolic dynamics.
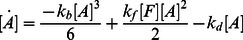
(3)


**Figure 1:**
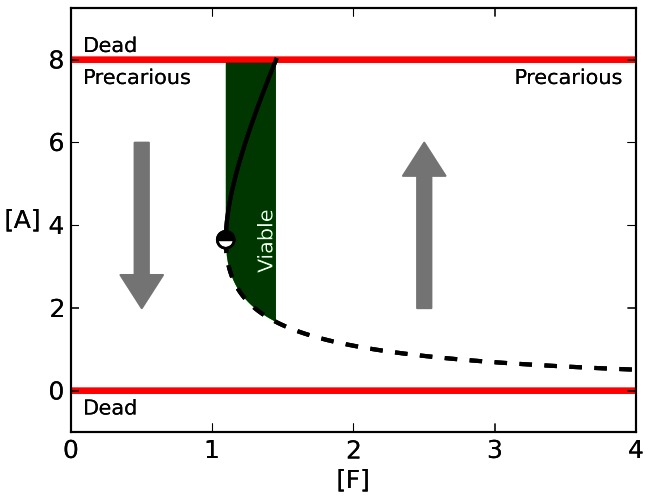
Equilibria, and the viable and precarious regions in the dynamical model of the metabolism. In the presented model, the system is only considered alive if 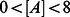. Given this constraint and the intrinsic metabolic dynamics described by Equation 3, two regions emerge: the precarious region (states for which 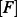 must be changed if the system is to persist) and the viable region (states for which no change in 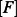 is necessary to avoid death).

The first and second terms represents the backward and forward reactions of Equation 1, and the third term represents the degradation of 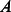. The stoichiometry of the reaction and its rate-constants (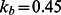, 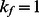, 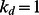) were assigned to produce a system that is bi-stable in the following sense: for certain fixed concentrations of 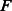, there is a stable ‘dead’ equilibrium at 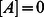, a stable viable equilibrium at 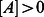 (indicated by the bold curve in Figure 1) and an unstable equilibrium (dashed curve). Piedrafita et al. [40] present a more detailed model of a metabolism that demonstrates similar bi-stable dynamics.

We impose one additional constraint upon the metabolism by including what we refer to as an “osmotic crisis” condition: if the concentration of 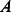 becomes too high, 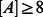, we say that the metabolism has grown too large causing the bacterium to burst. In the absence of this constraint, trivially simple strategies such as “grow 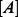 as fast as possible” or “maximize 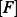
” accomplish optimal behavior and we wished to explore more sophisticated regulatory behavior that is capable of avoiding having too few resources and having too many - such as the oxygen-tactic behavior of *A. brasilense*, which avoids environments that have either too little or too much oxygen [41]. In modern bacteria, death by overfeeding may be rare or absent, here we consider how this regulation may be accomplished.

Figure 1 is a bifurcation plot that indicates the dynamics of the system that we have just described for different fixed concentrations of 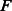. The “death” states are indicated by the red horizontal lines at 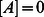 and 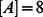. In between these lie a set of stable, viable equilibria (the bold curve) and a set of unstable equilibria (dashed curve). For states that are to the right of these curves, 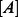 is increasing, and to the left of the curves, 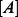 is decreasing (indicated by the arrows). As might be expected, when there is a low concentration of 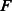, the system is incapable of self-producing at a rate sufficient to compensate for its degradation, resulting in a single stable equilibrium of no autocatalyst at 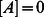. At 
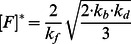
, the system bifurcates, and for a range of fixed concentrations 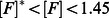, the system has three stable equilibria: a ‘viable’ equilibrium (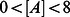), and two ‘dead’ equilibria (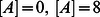). For fixed 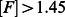, the system has only three possible final resting states: the two death states, and an unstable equilibrium in between. The set of states that are in the “viable region” are shaded in green, where for fixed 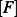, 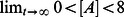, i. e. those states that end in a viable equilibrium. All other states fall within the “precarious region”: where 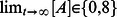, i. e. where in the absence of a change in 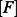, the system will fall into a “death” state. For further description of the notions of viable and precarious states and related concepts, see [35].

In this section, we have described the metabolic dynamics. The next section explains how we include different forms of behavior in the model, allowing us to compare their advantages and disadvantages.

### 2.2 Behavior

To compare different forms of behavior, we assume that the bacteria are in an unchanging environment and that they have some mechanism through which they can either increase or decrease 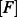 at some maximum rate (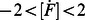). This change in 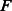 could be caused by motility such as the random “run/tumble” walk of *E. coli* and *R. sphaeroides*, which alternate between directed “running” and a random reorienting “tumbling” motion, modulating the frequency of tumbling to produce a stochastic motion toward regions of higher (or lower) chemical concentrations [4], [42]. Alternatively, the change in 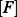 could be accomplished by the modulation of membrane properties or some other behavior but, for simplicity, we do not, at first, include these details in our model. Instead, we assume that each of the behavioral mechanisms that we examine can be described by an ordinary differential equation that describes how the behavior changes 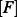. For each behavior, we evaluate the dynamics of the system specified by this “behavior” differential equation coupled with the “metabolism” differential equation (Equation 3). After our analysis of the behaviors using this abstract model, in Section 3.5 we present an extension of the model that includes spatial dynamics and chemotactic motility, and similar dynamics are observed.

We will consider four behaviors. Behavior 1 is the metabolism-independent regulation of the concentration of 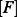. If the simulated bacterium is in an environment with resources less than the target concentration, it acts to increase their local concentration (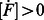), and if it is in an environment with too many resources, it behaves in some way to decrease their concentration (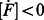). Behaviors 2 and 3 are both metabolism-based. Behavior 2 regulates the concentration of resources in response to the concentration of metabolic product, 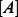. When 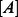 is lower than a target value, 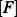 is increased, and when 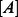 is higher than a target value, 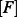 is decreased. Behavior 3 operates similarly, but instead of being a response to the concentration of metabolic product (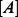), it is a response to the rate of change in the concentration of 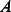: when 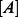 is decreasing, 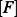 is increased and when 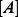 is increasing, 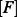 is decreased. The fourth and final behavior that we examine combines the first and third behaviors using a simple sigmoidal switching mechanism.

To allow for a fair comparison, for each behavior, we identified parameters that maximize survival-prolonging behavior. For Behaviors 1–3, there are only two parameters, and it was possible to thoroughly sample the parameter space (sampling 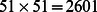 parameter pairs for each behavior) and identifying optimal parameter value-pairs (as evaluated by the fitness function described below). Behavior 4 has a 6-dimensional parameter space, making thorough search less feasible. We therefore employed the microbial genetic algorithm [43] to identify optimal parameters for this behavior. Table 1 lists the identified parametric values that were employed in our analysis.

**Table 1:** Parameters.

Parameter	Value	Behavior	Method of identification	Range
*θ_F_*	1.12	1	Lattice Sampling	[0..4]
*k_F_*	9.4	1	Lattice Sampling	[0..10]
*θ_A_*	4.0	2	Lattice Sampling	[0..8]
*k_A_*	10.0	2	Lattice Sampling	[0..10]
*θ_D_*	0.0	3	Lattice Sampling	[−2..2]
*k_D_*	10.0	3	Lattice Sampling	[0..10]
*θ_SF_*	1.1033	4	Genetic Algorithm	[0..4]
*k_SF_*	2.7381	4	Genetic Algorithm	[0..5]
*θ_SD_*	0.2894	4	Genetic Algorithm	[−1..1]
*k_SD_*	2.9981	4	Genetic Algorithm	[0..5]
*k_σ_*	2.6494	4	Genetic Algorithm	[0..4]
*k_r_*	6.0630	4	Genetic Algorithm	[0..10]

We used a variation of the microbial genetic algorithm [43] to identify optimal parameters for the switch-based behavior described in Section 3.4. This algorithm operates in the following way: a random population of 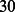 “genotypes” is generated. Each genotype specifies a set of the 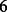 parameters for Behavior 4. This population is improved by repeatedly selecting two individuals at random from the population and comparing their “fitness” (their success at driving survival-prolonging behavior as evaluated by the fitness function described below) in what are called tournaments. The parameters of the less fit genotype, the “loser”, are made more similar to the parameters of the winner (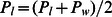), and then “mutated” -changed by a random amount selected from a Gaussian distribution with a mean of 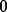 and a standard deviation that is one percent of the allowed range of values for that parameter. Parameters that are larger than the allowed range of values (see Table 1) after mutation “wrap around” via modular arithmetic to become low valued parameters and vice versa.

Each fitness evaluation involves running 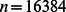 simulations for 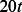, with initial conditions sampled from the space of 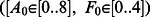 in a 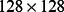 uniformly distributed rectangular lattice. Fitness 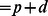, where 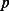 is the proportion of these simulations that are “survived” (i. e. where 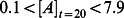) and 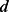 is the normalized mean distance of 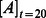 from 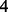 (the midpoint between the two dead states) as described by:
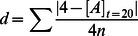
(4)


After 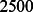 tournaments the population had converged on a highly fit set of parameters. We selected the fittest individual from the final population and used those parameters for our analysis. The parameters that were identified as optimal for all of the behaviors are presented in Table 1.

## Results

We shall now describe the four behaviors and the equations used to model them in detail. For each behavior we will analyze some example trajectories that provide insight into the dynamics of the coupled behavior-plus-metabolism system. We then evaluate the survivable initial conditions-those initial conditions that do not fall into a dead states after a long period of time. This analysis allows us to compare the different behaviors, identifying strengths and weaknesses of each.

### 3.1 Metabolism-independent Behavior

The first behavior that we shall consider is a metabolism-independent response to the environmental concentration of resources. This behavior is similar to metabolism-independent bacterial chemotaxis cases where the signaling pathways are not modulated by metabolic activity and are solely influenced by the concentration of resources in the environment. When resources are scarce, the bacteria act to increase the concentration of available resources and when resources are too high in concentration, the organism acts to decrease them. To model this, we use the following differential equation:
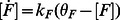
(5)


When 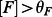, 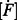 is negative, and when 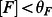, 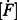 is positive, and the system thereby maintains 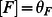. The parameter 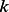 indicates the rate at which the system influences 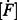, i. e. when 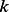 is higher, the system is more sensitive to the difference between 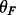 and 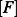. As mentioned in in Section 2.2, the values for these parameters (listed in Table 1) were selected to be the best possible parameters for maximizing survival prolonging behavior.

**Figure 2:**
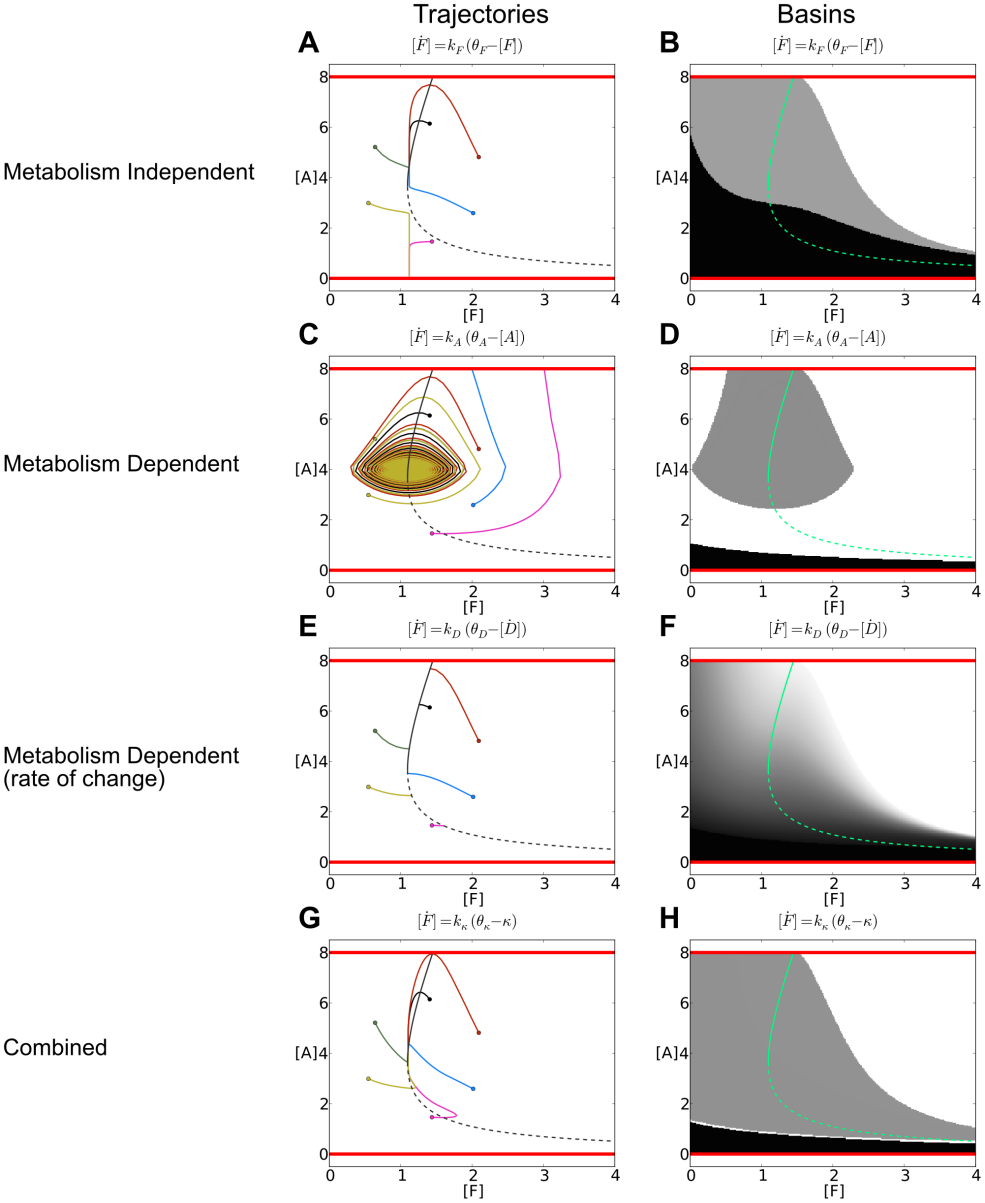
Example trajectories and survivable initial conditions for four different forms of behavior. Each of the left-hand plots indicate six example trajectories through the state space described in Section 2.1. The starting points for these trajectories (indicated by small, filled circles) are the same for each behavior. The right-hand plots indicate the “survivable initial conditions” for the same behaviors. The shade of gray on these graphs indicates 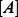 at 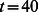 for the initial condition indicated by the location of the point on the plot. The white and black regions indicates initial conditions that end in the death states, 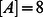 and 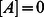 respectively. The mid-tones indicate initial conditions which survived. For all calculations, the system appeared to have come close to equilibrium before the end of the simulation.

The example trajectories plotted in Figure 2A provide insight into the advantages and disadvantages of this metabolism-independent behavior. Trajectories, like the green trajectory that starts near the upper-left, with 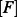 close to 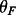, and 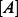 close to the viable equilibrium for 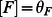, succeed at moving quickly to a stable equilibrium point within the viable region. However, when 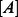 and/or 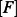 are too low or too high (e.g., the pink or yellow trajectories), the system dies. Some of these deaths are due to the rate of response; the simulated bacterium is appropriately increasing or decreasing 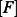, but it is not capable of doing so quickly enough (e. g. the yellow trajectory). Others are “making a mistake” in the sense that they are causing 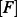 to change in the wrong direction, leading to death. For example, consider the the pink trajectory towards the lower right. The metabolism-independent behavior always causes a change in 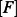 toward 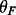, but in this case, this drives a suicidal behavior. If instead, 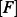 had increased for a short period of time, the system could have crossed the unstable equilibrium and then grown to a higher concentration of 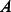. After some amount of growth, the homeostasis of 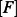 at 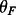 would have sufficed for survival.

The crucial point is that it does not suffice to respond only to 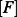, because there is no single concentration of 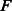 that is always the “right” concentration. For different values of 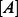, different concentrations of 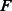 are needed to survive. Metabolism-independent behavior is, by definition, insensitive to the metabolic dimension of the situation, and so is incapable of driving survival prolonging behavior in some situations.

Figure 2B shows the final concentration of 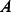 for different initial conditions, giving an impression of the strengths and weaknesses of this behavior. The white area indicates initial conditions that end at 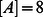, the osmotic crisis death, and the black area indicates initial conditions that result in a complete absence of metabolism, i. e. where 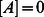. The mid-tones indicate approximations of the final concentration of 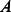 (i. e., 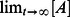), calculated by running numerical simulations for long enough that the system appears to come to equilibrium (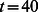). These mid-tones can be considered as the *survivable initial conditions* -those initial conditions for which the behavior succeeds at modulating 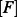 such that the system does not die.

### 3.2 Metabolism-based Behavior

Inspired by the metabolism-based chemotaxis of bacteria such as *E. coli*, *R. sphaeroides* and *Pseudomonads*, we shall now investigate a behavioral response to the concentration of metabolic product(s) rather than a direct response to environmental resources. The behavior works in the following way: when there is an excess of metabolic product (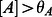), the behavior causes a decrease in available resources, slowing metabolite production. Conversely, when the concentration of metabolic product is low (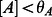), the behavior causes an increase in resources, thereby increasing the rate of metabolite production. We use the following equation to simulate this simple, but functional metabolism-based behavior.
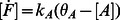
(6)


This equation has a similar form to the metabolism-independent mechanism presented above, but the behavior is now based on a sensitivity to the concentration of 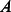 rather than of 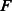. When 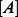 is higher than the target concentration 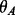, 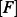 is decreased and when 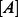 is lower, 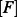 is increased. Decreasing 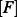 tends to decrease 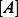, and so this behavior can result in a stable homeostasis of 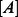 and 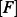.

Figure 2 c shows that this behavioral strategy can also be effective. Similar to the metabolism-independent mechanism described above, when initial conditions are close to the viable region, the system avoids the dead states, falling into an attractor within viable region (red, green, black and yellow trajectories). In fact, this metabolism-based behavior survives some of the initial conditions that the metabolism-independent behavior does not. For example, the initial condition of the yellow trajectory (lower-left) is survived by this behavior but it is not survived by the metabolism-independent behavior.

However, similar to the way that the metabolism-independent mechanism is blind to 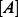, this behavior is, by definition, blind to 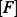. How to modulate 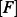 to drive survival-prolonging behavior is determined in part, by the state of 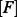, and therefore, like the metabolism-independent behavior, this behavior is incapable of driving survival-prolonging behavior in certain conditions. This limitation is apparent when we consider the pink (lower right) trajectory in Figure 2 c. Initially, the metabolism-based behavior appropriately increases 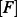. This increase continues until 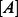 approaches 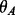. But at this point it is too late! There is so much 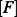 that the system will not be able to decrease 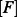 quickly enough to avoid osmotic crisis. Figure 2D shows the survivable initial conditions for this behavior, and it is apparent that similar problems occur when either 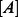 or 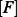 is quite low or high.

**Figure 3:**
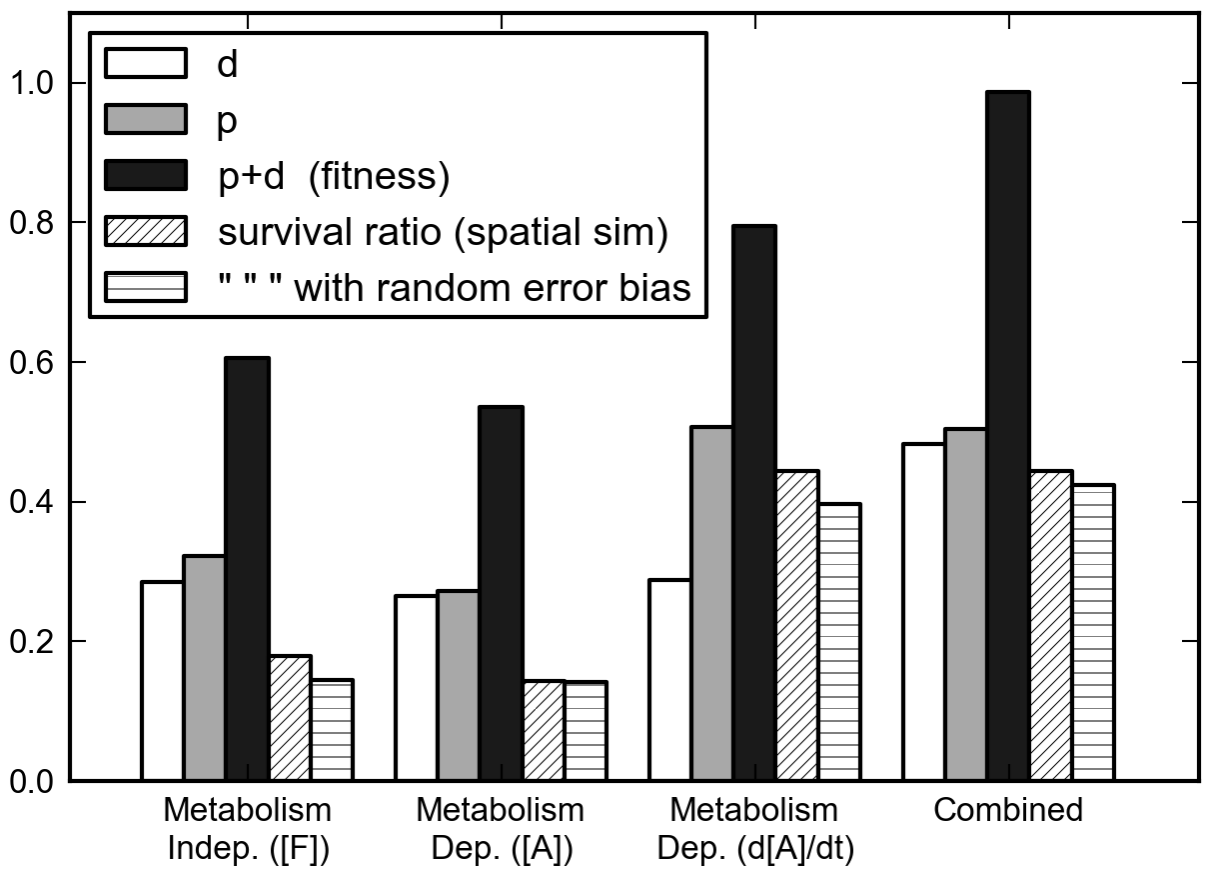
Evaluation of behaviors. The black columns indicate the fitness of each behavior as evaluated by the fitness function. Fitness is the sum of two terms: 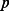, the proportion of tested initial conditions that are survived by the behavior and 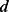, a normalized score that describes the mean distance of the final concentration of 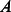 from the death states for all tested initial conditions (higher is better for all measurements). See Section 2.2 for further details of how these scores were calculated. Also plotted are the proportion of survival in the spatial simulations (see Section 3.5). Note that the spatial model survival rates follow a similar pattern to the fitness evaluation in the more abstract model.

So, although there are some initial conditions that this behavior survives that the metabolism-independent behavior does not, it is the metabolism-independent that survives the greater number of initial conditions. The gray columns in Figure 3 indicate the proportion of the 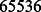 tested initial conditions that were survived by each behavior. For this model, the metabolism-independent behavior survives more initial-conditions and could therefore be arguably considered the more robust behavior. This result might be different for other models (if for instance, we chose different values for 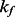, 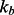, and/or 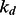), but as long as the relationship between the rate of metabolic growth, the concentration of metabolites and the concentration of metabolic resources is non-linear and there are upper and lower bounds on the viable resource levels, the central messages would remain the same: to drive survival-prolonging behavior in all conditions, it is insufficient to have only a simple behavioral response to either the metabolic product, or the concentration of environmental resources.

### 3.3 Metabolism-based Behavior 2: Responding to the Rate of Change

We now investigate a second form of metabolism-based behavior. Instead of responding to the concentration of metabolic product, this behavior is a response to the rate of change in the concentration of metabolic product. If the concentration of metabolic-product is changing in a way that is consistently positive or negative for an extended period of time, death will occur and it follows that a behavior might be able to avoid death by regulating resource availability to prevent change in the concentration of metabolic-product. This dynamic is captured by the following equation.
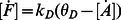
(7)


Again, the mechanism takes a similar form to those already described, except that now, instead of responding to 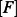 or 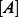, the behavior is in response to 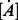. When 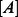 is increasing, 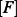 is reduced until 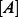 is no longer increasing. Similarly, if 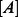 is decreasing, 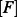 is increased until 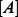 is no longer decreasing.

Figure 2E shows example trajectories for this behavioral mechanism. All of the selected example trajectories now avoid the two death boundaries, coming to rest on either the stable, viable equilibrium or the unstable equilibrium. The latter case (demonstrated by the yellow and pink trajectories) is an example of how behavior can stabilize inherently unstable metabolic dynamics, an idea that we are exploring in another paper under preparation.

This behavior survives more initial conditions than either of the previous behaviors (see Figures 2 and 3). Why should that be the case? The rate of metabolic growth is determined by both the concentration of metabolites and the amount of resources available. This response is therefore, indirectly sensitive to both the (metabolism-based) concentration of metabolites *and* the (metabolism-independent) concentration of metabolic resources, meaning that unlike the other two behaviors, this one is influenced both by 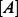 and 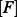, allowing for a greater range of survivable conditions.

Note however, that some of end-states have a concentration of 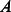 that is close to death meaning that a small fluctuation in 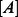 could result in death. Also, some trajectories end on a saddle-node point where 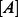 is unstable and biased fluctuations could bring the system increasingly close to 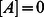. So, although more initial conditions are survived, some of the equilibria found by this behavior would not be as robust to perturbations as the equilibria of previous behaviors. The white columns in Figure 3 indicate the mean distance of the final 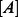 from death states, showing that this behavior does not, on average, keep the system much farther away from death than the other two behaviors. In some respects, this behavior appears better than the two previous, but as we shall show in the next section, an improvement is possible through combining metabolism-independent and metabolism-based behaviors.

### 3.4 Switching between Metabolism-based and Metabolism-independent Sensitivities

All of the basic forms of behavior that we have examined, whether metabolism-independent or metabolism-based, have been insufficient to drive survival-prolonging behavior in certain survivable initial conditions. The “rate-of-change” mechanism (Section 3.3) is capable of surviving a greater number of initial conditions than the others, but as mentioned, some final equilibria are quite precarious. A range of empirical bacterial chemotaxis research suggests that metabolism-based and metabolism-independent mechanisms may operate concurrently within *E. coli*, *R. sphaeroides* and *Pseudomonads*. We postulate that a simple switching mechanism is sufficient to integrate the two different forms of behavioral sensitivity in a way that is beneficial. One way to model this is to introduce a simple smooth sigmoidal switch that responds to 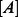 such that when 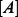 is high, the 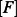-sensitivity described in Section 3.1 is the dominant mechanism, and when 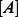 is low, the metabolism-based derivative mechanism that we explored in Section 3.3 is the dominant mechanism.

The equations that we use to model this behavior are the following.
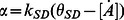
(8)

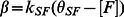
(9)

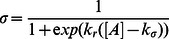
(10)

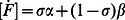
(11)


In these equations, 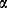 describes the influence of the metabolism-based “rate-of-change” mechanism described in 3.3, 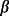 describes the influence of the metabolism-independent mechanism described in 3.1, and 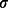 represents the state of the switch, which attenuates 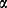 and 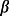 in response to 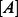 (as described by Equations 10 and 11). Thus, when 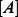 is below 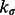, 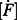 is predominantly influenced by the metabolism-based “rate-of-change” sub-mechanism, and when 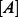 is above 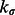, the predominant influence is a response to 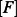, similar to that described in Section 3.1. The parameter 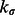 describes the point where the two mechanisms are equally influential and 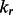 determines the smoothness of the sigmoidal function. As mentioned above, the parameters for this behavior were identified by using a genetic algorithm to produce behavior that avoids the death equilibria (see Section 2.2).

Figures 2G and 2H show example trajectories and the survivable initial conditions for this behavioral mechanism. As with the rate-of-change mechanism, all of the example trajectories manage to avoid death, but now they all end in a less precarious equilibrium, in the middle of the viable region. This behavior is therefore the most effective of those that we have evaluated, providing theoretical support for the evidence of concurrent mechanisms metabolism-based and metabolism-independent mechanisms operating in various bacteria. It is likely that the mechanism of integration in bacteria is complicated, but here we have demonstrated that even a simple switching mechanism that responds to the concentration of a metabolic product can be sufficient to benefit from concurrent metabolism-based and metabolism-independent sensitivities.

### 3.5 Different Behavioral Mechanisms Produce Different Spatio-temporal Patterns

In this section we present a spatial simulation of bacteria performing chemotaxis driven by each of the four different behaviors described above. In this spatial model, we simulate the metabolism of 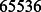 bacteria, but instead of having the behavioral mechanism directly influence the concentration of 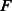, we consider the bacteria to be spatially embedded in a one-dimensional environment, in which there is a fixed gradient of 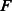. The location of each bacterium 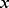, determines 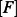 according to the following formula.
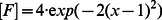
(12)


Provided that they are “alive” (defined by 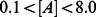), the simulated bacteria are always either moving up-gradient (toward increasingly positive 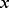) or down-gradient. The direction of the movement is determined by the sign of 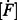 as determined by the relevant behavior. A negative 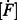 causes a down-gradient motion and a non-negative 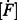 causes up-gradient motion according to the following piece-wise differential equation, where 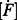 is the change in 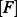 as specified by the behavioral mechanism that is being investigated, and 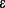 is a randomly assigned fixed error bias term that is described below.
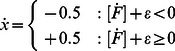
(13)


This environment has “walls” in the sense that any bacterium with 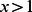 are immediately relocated to 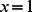 and similarly those with 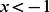 are relocated to 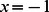.

**Figure 4:**
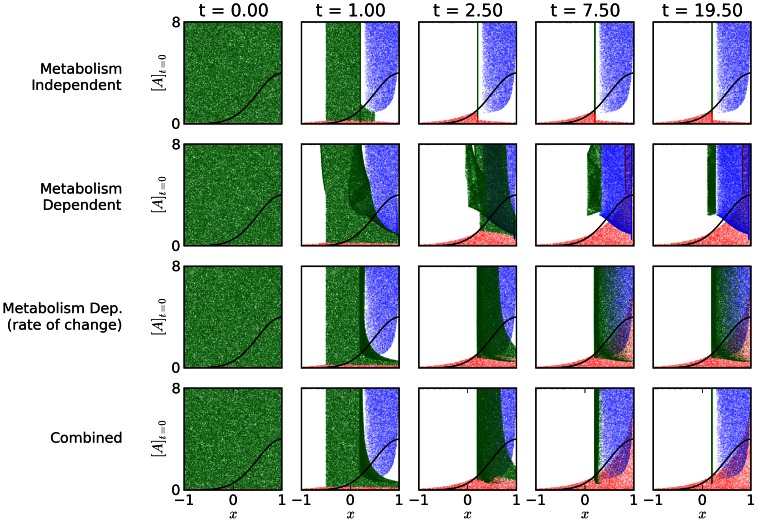
Spatio-temporal distributions of bacteria performing the four different behaviors. Spatial location (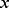) plotted against the initial metabolic state of the simulated bacteria 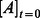. The distribution of 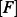 in the environment is indicated by the black curve. Blue points indicate bacteria that have died due to excess (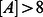), red points indicate bacteria that have died due to running low of autocatalyst (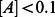), and green points indicated bacteria that are alive (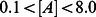). The four behaviors produce qualitatively different spatio-temporal distributions.

At the start of the simulation, the location of each bacterium is selected from an flat distribution covering the range between the two walls 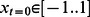. To investigate how the state of the metabolism influences subsequent behavior, the initial metabolic states of the simulated bacteria are selected from a flat distribution 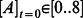. It may be possible to similarly vary the initial metabolic state of bacteria in a chemotaxis assay through starving or over-feeding bacteria before observing their chemotaxis in a gradient environment. Figure 4 shows the temporal evolution of the spatial distributions of bacteria performing the different behaviors, when the error term 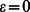. Each point indicates the spatial location of the bacteria (horizontal axis), plotted against its initial metabolic state, 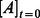 (vertical axis). The colors of the points indicate the living/dead status of the bacterium, with blue points indicating bacteria that have died due to excess 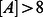, red points indicating bacteria that have died due to insufficient autocatalyst 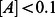, and green points indicated bacteria that are alive. Animations of these plots are available at http://www.youtube.com/watch?v=h68pBQ8alns.

In all cases, regardless of the behavior simulated, a large proportion of the bacteria perform chemotaxis to a region partway up the resource gradient where 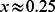 and 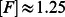. However, the four behaviors do so in different ways, producing different spatio-temporal distributions. In the metabolism-independent behavior, different initial values of 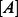 cause no difference in the behavior of the bacteria, and all bacteria move toward 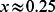, with some dying along the way, depending on initial conditions. The first metabolism-based behavior drives an oscillating response where the bacteria move toward 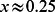, overshoot it, turn back toward it, overshoot it again etc. The “rate-of-change” metabolism-based mechanism drives chemotaxis such that those bacteria that start far down-gradient of 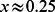 with low 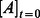 move up-gradient to 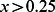 and remain there. The insight provided by our analysis in Section 3.3 and in Figure 2 suggests that these bacteria have stopped in an area where the concentration of 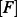 is matched with their (relatively low) 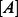, such that 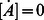. The combined switching behavior similarly compensates for low 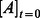 by moving to 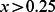, but in a way that is different from the rate-of-change mechanism. For this behavior, 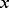 is increased such that 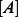 increases. After some increase in 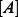, the metabolism-independent part of its behavior dominates, and the bacteria move down-gradient to 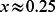.

The bar-chart in Figure 3 includes the proportion of these simulated bacteria that survived(i. e., for which 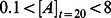) in each of these four simulations. Also included in this figure are the ratios of survival for spatial simulations where the 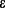 for each simulated bacterium was selected from a normal distribution with a mean of 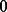 and a standard deviation of 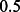. These results support the more abstract non-spatial analysis conducted in previous sections, with the combined behavior and rate-of-change behaviors allowing for survival in a greater range of situations than the other two behaviors, and the combined-mechanism suffering slightly less from the error bias than the rate-of-change mechanism.

## Discussion

Experimental work has shown that the bacterial chemotaxis is sometimes metabolism-independent, sometimes metabolism-based, and that certain bacteria appear to utilize both of these forms of behavior. Each mechanism has advantages and disadvantages. For example, metabolism-based responses are inherently capable of integrating simultaneous environmental influences into an appropriate response [33] while metabolism-independent mechanisms allow bacteria to respond to trace quantities of attractants that are too low in concentration to affect on the metabolism, but are nevertheless good indicators of where an organism could find more resources. The mechanisms are different, but neither is clearly superior to the other. Here we have elaborated upon these differences by presenting a dynamical analysis of different forms of basic metabolism-based and metabolism-independent behavior in a minimal model. The model has made clear that basic forms of regulation, whether metabolism-based or metabolism-independent, are insufficient to drive survival prolonging behavior in certain survivable situations. This is true because the rate of metabolic growth is influenced both by the state of the metabolism and by the state of the environment. The appropriate behavioral modulation of the environmental state therefore also depends on the state of the metabolism and the state of the environment, and purely metabolism-based or metabolism-independent mechanisms are, by definition, blind to one of these dimensions. We suggest that this conclusion holds in the vast majority of cases, provided that there is a non-linear relationship between resources, the state of the metabolism and the rate of metabolic growth. It may be possible that more sophisticated forms of metabolism-based or metabolism-based behavior are able to drive more successful behavior, and we are currently exploring this possibility.

In our model, a mechanism that switches between metabolism-based and metabolism-independent sensitivities was capable of driving survival-prolonging behavior in more conditions than any of the basic metabolism-independent or metabolism-based behaviors, demonstrating that a basic switching device suffices to integrate the two forms of behavior in a beneficial way. This raises the hypothesis that a switching mechanism could be in operation within bacteria that appear to have both metabolism-based and metabolism-independent chemotactic mechanisms. However, real metabolisms are much more complicated, and the relationship between the metabolism and its environment is likely to be more complex, leading us to expect a rich interaction between metabolism-independent and metabolism-based sensitivities. We also showed that the rate-of-change in the concentration of metabolic product can act as an indirect indicator of both the state of the metabolism and the state of available metabolic resources, allowing for an improvement over mechanisms that respond only to the concentration of available metabolic resources or the concentration of metabolic product. We are currently investigating other, more sophisticated forms of regulation that are based on a response to the rate-of-change of metabolic product.

In Section 3.5, we presented a spatial simulation of bacteria with motile behavior driven by the different behavioral mechanisms. These simulations supported the conclusions of the dynamical analysis of our more abstract model presented earlier in the paper, and they also demonstrated that each of the four different behavioral forms investigated produce different spatio-temporal distributions of bacteria. All but one of these are influenced by the initial metabolic state of the bacteria and we suggest that it may be possible to experimentally derive insight into the relationship between metabolism and chemotaxis in real bacteria, by experimentally varying the state of their metabolism before placing them on a resource gradient and observing how their spatial distribution changes over time. Given the complexity of metabolism and behavioral mechanisms in modern bacteria, it is highly unlikely that the patterns in our abstract simulation will directly correspond to patterns observed in experimental chemotaxis assays. Nevertheless, it is our hope that our analysis of the mechanisms at play in our minimal model will help to interpret the patterns observed in chemotactic bacteria.

More broadly, we have started here (and in our other recent work) an investigation into to the role of somatic processes in sensorimotor loops. If we are ever to completely understand chemotaxis and other, more complicated forms of behavior, it may be necessary to include details of metabolic and other somatic processes in our analysis.
